# *N*′-[(*E*)-3-Pyridylmethyl­idene]benzo­hydrazide

**DOI:** 10.1107/S160053680903894X

**Published:** 2009-10-03

**Authors:** Liyuan Wen, Handong Yin, Wenkuan Li, Kang Li

**Affiliations:** a College of Chemistry and Chemical Engineering, Liaocheng University, Shandong 252059, People’s Republic of China

## Abstract

The title compound, C_13_H_11_N_3_O, was prepared by the reaction of benzohydrazide and nicotinaldehyde. The dihedral angle between the planes of the two aromatic rings is 47.78 (9)°. The crystal structure is stabilized by inter­molecular N—H⋯N hydrogen-bonding inter­actions.

## Related literature

For related structures, see: Yin *et al.* (2008 ▶).
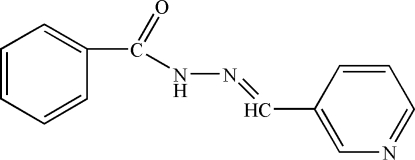


## Experimental

### 

#### Crystal data

C_13_H_11_N_3_O
*M* *_r_* = 225.25
Orthorhombic, 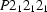

*a* = 7.6193 (13) Å
*b* = 10.6291 (17) Å
*c* = 13.530 (2) Å
*V* = 1095.7 (3) Å^3^
*Z* = 4
Mo *K*α radiation
μ = 0.09 mm^−1^
*T* = 298 K
0.21 × 0.18 × 0.08 mm

#### Data collection

Siemens SMART CCD diffractometer
Absorption correction: multi-scan (*SADABS*; Sheldrick, 1996 ▶) *T*_min_ = 0.981, *T*_max_ = 0.993
5473 measured reflections
1136 independent reflections
612 reflections with *I* > 2σ(*I*)
*R*_int_ = 0.073

#### Refinement

*R*[*F* ^2^ > 2σ(*F* ^2^)] = 0.042
*wR*(*F* ^2^) = 0.104
*S* = 1.18
1136 reflections
154 parameters
H-atom parameters constrained
Δρ_max_ = 0.20 e Å^−3^
Δρ_min_ = −0.20 e Å^−3^

### 

Data collection: *SMART* (Siemens, 1996 ▶); cell refinement: *SAINT* (Siemens, 1996 ▶); data reduction: *SAINT*; program(s) used to solve structure: *SHELXS97* (Sheldrick, 2008 ▶); program(s) used to refine structure: *SHELXL97* (Sheldrick, 2008 ▶); molecular graphics: *SHELXTL* (Sheldrick, 2008 ▶); software used to prepare material for publication: *SHELXTL*.

## Supplementary Material

Crystal structure: contains datablocks I, global. DOI: 10.1107/S160053680903894X/gk2228sup1.cif

Structure factors: contains datablocks I. DOI: 10.1107/S160053680903894X/gk2228Isup2.hkl

Additional supplementary materials:  crystallographic information; 3D view; checkCIF report

## Figures and Tables

**Table 1:** Hydrogen-bond geometry (Å, °)

*D*—H⋯*A*	*D*—H	H⋯*A*	*D*⋯*A*	*D*—H⋯*A*
N1—H1⋯N3^i^	0.86	2.40	3.236 (5)	164

Symmetry code: (i) 
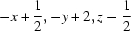
.

**Fig. 1.**
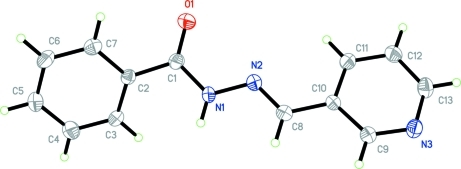
The molecule of the title compound, shown with 50% probability displacement ellipsoids.

**Fig. 2.**
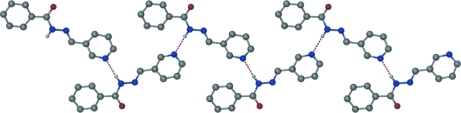
A view of the one-dimensional extended chain structure in the title compound.

## supplementary crystallographic information

### Comment

Acylhydrazones, as an example of Schiff bases, and their metal complexes have been widely studied due to their versatile applications in the fields of analytical and medicinal chemistry and biotechnology. These ligands, owing to their facile keto-enol tautomerization and the availability of several potential donor sites, can coordinate with metals (Yin *et al.*, 2008). We report here the synthesis and structure of the title compound. The molecular structure of the title compound is shown in Fig. 1. The hydrazone molecule crystallizes as an E isomer. In the crystal structure, there exist intermolecular N—H···N hydrogen bonds (Table 1). As seen in Fig. 2, the molecules are linked into one-dimensional extended chain structure.

### Experimental

A mixture of benzohydrazide (10 mmol) and nicotinaldehyde (10 mmol) was refluxed in ethanol (40 ml) for 2 h at 353K. After the solution had cooled down to room temperature yellow sediment appeared. The product was crystallized from a solution of methanol to yield yellow block-shaped crystals of the title compound (yield 78%). Anal. Calcd (%) for C_13_H_11_N_3_O (Mr = 225.25): C,69.32; H, 4.92; N, 18.65. Found (%): C, 69.21; H, 4.97; N, 18.76.

### Refinement

In the absence of significant anomalous scattering effects, Friedel pairs were averaged. The C—H and N—H H atoms were positioned with idealized geometry (N—H = 0.86 Å and C—H = 0.93 Å) and were refined using a riding model approximation with *U*_iso_(H) = 1.2 *U*_eq_(C, N).

### Crystal data

**Table:**

C_13_H_11_N_3_O	*F*(000) = 472
*M**_r_* = 225.25	*D*_x_ = 1.365 Mg m^−^^3^
Orthorhombic, *P*2_1_2_1_2_1_	Mo *K*α radiation, λ = 0.71073 Å
Hall symbol: P 2ac 2ab	Cell parameters from 764 reflections
*a* = 7.6193 (13) Å	θ = 2.4–25.1°
*b* = 10.6291 (17) Å	µ = 0.09 mm^−^^1^
*c* = 13.530 (2) Å	*T* = 298 K
*V* = 1095.7 (3) Å^3^	Block, yellow
*Z* = 4	0.21 × 0.18 × 0.08 mm

### Data collection

**Table:**

Siemens SMART CCD diffractometer	1136 independent reflections
Radiation source: fine-focus sealed tube	612 reflections with *I* > 2σ(*I*)
graphite	*R*_int_ = 0.073
φ and ω scans	θ_max_ = 25.0°, θ_min_ = 2.4°
Absorption correction: multi-scan (*SADABS*; Sheldrick, 1996)	*h* = −9→8
*T*_min_ = 0.981, *T*_max_ = 0.993	*k* = −12→11
5473 measured reflections	*l* = −12→16

### Refinement

**Table:**

Refinement on *F*^2^	Primary atom site location: structure-invariant direct methods
Least-squares matrix: full	Secondary atom site location: difference Fourier map
*R*[*F*^2^ > 2σ(*F*^2^)] = 0.042	Hydrogen site location: inferred from neighbouring sites
*wR*(*F*^2^) = 0.104	H-atom parameters constrained
*S* = 1.18	*w* = 1/[σ^2^(*F*_o_^2^) + (0.0242*P*)^2^ + 0.2399*P*] where *P* = (*F*_o_^2^ + 2*F*_c_^2^)/3
1136 reflections	(Δ/σ)_max_ < 0.001
154 parameters	Δρ_max_ = 0.20 e Å^−^^3^
0 restraints	Δρ_min_ = −0.20 e Å^−^^3^

### Fractional atomic coordinates and isotropic or equivalent isotropic displacement parameters (Å^2^)

**Table:**

	*x*	*y*	*z*	*U*_iso_*/*U*_eq_	
N1	0.1276 (5)	0.7488 (3)	0.4106 (3)	0.0430 (12)	
H1	0.1551	0.8129	0.3750	0.052*	
N2	0.1085 (5)	0.7596 (4)	0.5111 (3)	0.0410 (11)	
N3	0.1881 (5)	1.0185 (4)	0.7966 (3)	0.0450 (12)	
O1	0.0701 (5)	0.5389 (3)	0.4181 (2)	0.0544 (10)	
C1	0.1015 (7)	0.6334 (4)	0.3688 (4)	0.0386 (13)	
C2	0.1109 (6)	0.6291 (4)	0.2602 (3)	0.0327 (12)	
C3	0.0535 (7)	0.7257 (4)	0.2001 (4)	0.0438 (14)	
H3	0.0107	0.7992	0.2285	0.053*	
C4	0.0588 (7)	0.7150 (5)	0.0986 (4)	0.0523 (15)	
H4	0.0175	0.7805	0.0594	0.063*	
C5	0.1247 (7)	0.6078 (5)	0.0548 (4)	0.0559 (17)	
H5	0.1301	0.6013	−0.0137	0.067*	
C6	0.1826 (7)	0.5105 (4)	0.1136 (4)	0.0515 (15)	
H6	0.2268	0.4376	0.0848	0.062*	
C7	0.1748 (6)	0.5212 (4)	0.2156 (4)	0.0448 (14)	
H7	0.2132	0.4547	0.2547	0.054*	
C8	0.1525 (6)	0.8640 (5)	0.5499 (3)	0.0446 (14)	
H8	0.1941	0.9288	0.5101	0.054*	
C9	0.1968 (6)	0.9926 (4)	0.6998 (3)	0.0432 (14)	
H9	0.2460	1.0533	0.6588	0.052*	
C10	0.1383 (6)	0.8825 (4)	0.6558 (4)	0.0365 (13)	
C11	0.0676 (6)	0.7933 (4)	0.7186 (4)	0.0416 (14)	
H11	0.0272	0.7173	0.6933	0.050*	
C12	0.0567 (7)	0.8168 (5)	0.8183 (4)	0.0492 (15)	
H12	0.0094	0.7572	0.8610	0.059*	
C13	0.1173 (6)	0.9306 (5)	0.8537 (4)	0.0497 (15)	
H13	0.1080	0.9465	0.9211	0.060*	

### Atomic displacement parameters (Å^2^)

**Table:**

	*U*^11^	*U*^22^	*U*^33^	*U*^12^	*U*^13^	*U*^23^
N1	0.066 (3)	0.036 (2)	0.026 (2)	−0.006 (2)	0.001 (2)	−0.0031 (19)
N2	0.051 (3)	0.039 (2)	0.033 (3)	−0.002 (2)	0.003 (2)	0.0001 (19)
N3	0.048 (3)	0.046 (2)	0.041 (3)	−0.003 (2)	0.001 (2)	−0.008 (2)
O1	0.080 (3)	0.0399 (19)	0.044 (2)	−0.009 (2)	−0.004 (2)	0.0059 (18)
C1	0.043 (3)	0.036 (3)	0.037 (3)	−0.005 (3)	−0.006 (3)	−0.003 (3)
C2	0.030 (3)	0.033 (3)	0.035 (3)	−0.004 (3)	0.002 (3)	−0.004 (2)
C3	0.057 (4)	0.033 (3)	0.042 (4)	0.004 (3)	0.005 (3)	−0.005 (3)
C4	0.063 (4)	0.054 (3)	0.040 (4)	−0.002 (3)	−0.007 (3)	0.003 (3)
C5	0.077 (4)	0.056 (4)	0.035 (3)	−0.002 (3)	0.002 (3)	−0.006 (3)
C6	0.063 (4)	0.037 (3)	0.055 (4)	0.004 (3)	0.005 (3)	−0.011 (3)
C7	0.050 (4)	0.036 (3)	0.049 (4)	−0.004 (3)	0.000 (3)	−0.001 (3)
C8	0.057 (4)	0.039 (3)	0.038 (3)	−0.003 (3)	0.001 (3)	0.003 (3)
C9	0.056 (4)	0.038 (3)	0.036 (3)	−0.002 (3)	0.002 (3)	0.000 (3)
C10	0.044 (3)	0.035 (3)	0.030 (3)	−0.002 (3)	0.000 (3)	0.002 (2)
C11	0.045 (4)	0.038 (3)	0.042 (4)	−0.002 (3)	0.000 (3)	−0.002 (3)
C12	0.060 (4)	0.048 (3)	0.039 (3)	−0.010 (3)	0.004 (3)	0.006 (3)
C13	0.052 (4)	0.061 (3)	0.037 (3)	−0.002 (3)	0.004 (3)	−0.006 (3)

### Geometric parameters (Å, °)

**Table:**

N1—C1	1.365 (5)	C5—H5	0.9300
N1—N2	1.372 (5)	C6—C7	1.386 (6)
N1—H1	0.8600	C6—H6	0.9300
N2—C8	1.273 (6)	C7—H7	0.9300
N3—C13	1.327 (6)	C8—C10	1.451 (6)
N3—C9	1.340 (5)	C8—H8	0.9300
O1—C1	1.229 (5)	C9—C10	1.386 (6)
C1—C2	1.473 (6)	C9—H9	0.9300
C2—C3	1.380 (6)	C10—C11	1.381 (6)
C2—C7	1.385 (6)	C11—C12	1.375 (6)
C3—C4	1.378 (6)	C11—H11	0.9300
C3—H3	0.9300	C12—C13	1.381 (6)
C4—C5	1.379 (6)	C12—H12	0.9300
C4—H4	0.9300	C13—H13	0.9300
C5—C6	1.377 (6)		
			
C1—N1—N2	118.1 (4)	C7—C6—H6	120.0
C1—N1—H1	121.0	C2—C7—C6	121.1 (5)
N2—N1—H1	121.0	C2—C7—H7	119.5
C8—N2—N1	117.0 (4)	C6—C7—H7	119.5
C13—N3—C9	116.4 (4)	N2—C8—C10	120.4 (5)
O1—C1—N1	122.5 (5)	N2—C8—H8	119.8
O1—C1—C2	121.7 (5)	C10—C8—H8	119.8
N1—C1—C2	115.7 (4)	N3—C9—C10	125.2 (4)
C3—C2—C7	118.1 (4)	N3—C9—H9	117.4
C3—C2—C1	123.4 (5)	C10—C9—H9	117.4
C7—C2—C1	118.5 (5)	C11—C10—C9	116.2 (4)
C4—C3—C2	121.1 (5)	C11—C10—C8	122.9 (5)
C4—C3—H3	119.5	C9—C10—C8	120.9 (5)
C2—C3—H3	119.5	C12—C11—C10	120.1 (5)
C3—C4—C5	120.5 (5)	C12—C11—H11	119.9
C3—C4—H4	119.8	C10—C11—H11	119.9
C5—C4—H4	119.8	C11—C12—C13	118.6 (5)
C6—C5—C4	119.2 (5)	C11—C12—H12	120.7
C6—C5—H5	120.4	C13—C12—H12	120.7
C4—C5—H5	120.4	N3—C13—C12	123.4 (5)
C5—C6—C7	120.0 (5)	N3—C13—H13	118.3
C5—C6—H6	120.0	C12—C13—H13	118.3

### Hydrogen-bond geometry (Å, °)

**Table:**

*D*—H···*A*	*D*—H	H···*A*	*D*···*A*	*D*—H···*A*
N1—H1···N3^i^	0.86	2.40	3.236 (5)	164

Symmetry codes: (i) −*x*+1/2, −*y*+2, *z*−1/2.
